# Renoprotective Effects of *Vitex megapotamica* (Spreng.) Moldenke in C57BL/6 LDLr-Null Mice Undergoing High Fat Diet

**DOI:** 10.1155/2015/475380

**Published:** 2015-02-19

**Authors:** Valdinei de Oliveira Araújo, Francielly Mourão Gasparotto, Vanessa Aranega Pires, Aline Antunes Maciel, Caroline Flach Ortmann, Euclides Lara Cardozo Junior, Emerson Luiz Botelho Lourenço, Arquimedes Gasparotto Junior

**Affiliations:** ^1^Instituto de Ciências Biológicas, Médicas e da Saúde, Universidade Paranaense, P.O. Box 224, 87.502-210 Umuarama, PR, Brazil; ^2^Laboratório de Farmacologia Cardiovascular, Faculdade de Ciências da Saúde, Universidade Federal da Grande Dourados, Rodovia Dourados, Itahum, Km 12, P.O. Box 533, 79.804-970 Dourados, MS, Brazil; ^3^Departamento de Ciências Farmacêuticas, Universidade Federal de Santa Catarina, Rua Roberto Sampaio Gonzaga, 88.040-380 Florianópolis, SC, Brazil

## Abstract

Although* Vitex megapotamica* (Spreng.) Moldenke is used in Brazilian folk medicine as hypolipidemic drug no study has been conducted to evaluate the effects of this species in an experimental model of atherosclerosis. So, the aim of this study was to evaluate the possible renoprotective activity of methanolic extract obtained from* Vitex megapotamica* (MEVM) using C57BL/6 LDLr-null mice submitted to high fat diet (HFD). MEVM was orally administered at doses of 30, 100, and 300 mg/kg, for three weeks, starting from the 2nd week of HFD. Systolic blood pressure (SBP) and diuretic activity were measured weekly. At the end of experiments the serum lipids, atherogenic index serum (AIS), oxidative stress, and markers of renal function were determined. HFD induced a significant increase in the systolic blood pressure, dyslipidemia, increase in AIS, and lipid peroxidation accompanied by an important reduction in renal function. Treatment with MEVM was able to prevent increase in SBP, total cholesterol, triglycerides, AIS, urea, and creatinine levels in LDLr-null mice. These effects were accompanied by a significant reduction in oxidative stress and renal injury. The data reported here support the potential of* Vitex megapotamica* as candidate to be an herbal medicine used in cardiovascular or renal diseases.

## 1. Introduction

Atherosclerosis is a process that consists of chronic and progressive alterations in arterial wall characterized by inflammatory and fibroproliferative response. Endothelial dysfunction appears to be the initial step, and dyslipidemia, often caused by high fat diet (HFD), is one of its main triggers. The atherosclerotic lesion may affect several important arterial territories, including renal arteries and abdominal aorta, being an important factor in the development of renal insufficiency (RI). Furthermore, it is noteworthy that kidney, an organ with wide vasculature, could be a sophisticated sensor of subclinical cardiovascular damage induced by endothelial injury [1].

Despite its proven effectiveness, lifestyle changes and hypolipidemic drugs apparently did not prevent RI evolution in all dyslipidemic patients, possibly due to genetic characteristics and/or other comorbidities. Furthermore, the elevated prevalence of adverse effects and poor patient compliance to treatment has reduced its efficacy [2]. In Brazil, the use of natural products to treat cardiovascular and renal diseases is very expressive, and considering the high biodiversity of plants in Brazil, several species have been very poorly investigated, mainly for renal function disorders [3].

*Vitex megapotamica* (Spreng.) Moldenke (Lamiaceae) occurs in the Atlantic Forest environment, from Bahia down to Rio Grande do Sul, and also in Florestas de Pinhais and Florestas Semideciduas of the Paraná River basin [4]. It is popularly known as Tarumã (*Tupi-Guarani*—“dark fruit to make wine”), olives, and olive-brave-the-ground. In folk medicine, the infusion of the leaves of this plant is used in the treatment of hemorrhoids, hypertension, and as diuretic, hypocholesterolemic, anti-inflammatory, among other therapeutic properties [5, 6]. This species has been characterized due to the presence of various substances of pharmacological interest, such as tannins, flavonoid glycosides, polyphenols, alkaloids, saponins, and essential oils [7, 8].

Few pharmacological and toxicological studies have been carried out with this species. Brandt et al. [7] have shown hypolipidemic effect by the decrease of serum cholesterol and triacylglycerides levels in mice after administration of the ethanolic extract and decoction obtained from* Vitex megapotamica*. Furthermore, two toxicological preliminary assays have shown no mutagenic activity or damage in cardiac and hepatic tissues, as well as in kidney physiology [7, 8].

Despite information about the cardiovascular and renal benefits of this species and its extensive folk use as antihypertensive, diuretic, and hypolipidemic, there are no published data showing its impact on the prevention and/or progression of renal disorders during dyslipidemia and atherosclerosis. Therefore, the aim of this study was to evaluate possible diuretic effects and renoprotective activity of the methanolic extract obtained from* Vitex megapotamica* using C57BL/6 LDLr-null mice submitted to high fat diet (HFD).

## 2. Materials and Methods

### 2.1. Drugs

Simvastatin, Fast Blue BB salt, Folin-Ciocalteu's phenol reagent, and gallic acid were obtained from Sigma-Aldrich Chemical Co. (St. Louis, MO, USA). HPLC reagents were purchased from Tedia (Brazil) and Vetec (Brazil). All other reagents and drugs used in this study were acquired with analytical grade.

### 2.2. Plant Material

Leaves were obtained from plants growing in an experimental botanical garden and authenticated as* Vitex megapotamica.* The cultivation is located at an altitude of 620–650 m under the following coordinates: S 25°03′28′′–W 53°52′37′′, in the municipality of Vera Cruz do Oeste, PR, Brazil. A voucher specimen (Selusniaki M 224) was deposited in the herbarium at the “*Museu Botânico Municipal de Curitiba (MBM)*”.

### 2.3. Preparation of the Methanolic Extract

*Vitex megapotamica* leaves were dried in an oven with forced air circulation (45°C, 48 h). Subsequently, leaves were crushed (Metvisa, mod. LQL.4). Crude extract was prepared by maceration (1 : 5 w/v) from methanol/water (70 : 30 v/v) at room temperature for fifteen days. After this period, preparations were filtered and concentrated under negative pressure (−750 mmHg) at 50°C in rotary evaporator (Fisatom, mod. 801). After removing the organic solvent, the concentrated extract was dried in lyophilizer, labeled, and stored at −20°C. The lyophilized methanolic extract (MEVM) was used to perform the phytochemical analysis and evaluate the pharmacological activity.

### 2.4. Phytochemical Analysis

#### 2.4.1. Screening

An aliquot of extract was used in the phytochemical screening, which was performed using methodology adapted by Brant et al. (2014) [9]. We investigated the presence of the following chemical groups: coumarins, polyphenols, tannins, catechins, flavonoids, anthocyanins and anthraquinones, alkaloids, methylxanthines, and saponins. Total phenols were measured by Folin-Ciocalteu and Fast Blue BB method [10], and the results were expressed as gallic acid equivalents (GAE), *y* = 0.0039*x* − 0.1152, *R* ^2^ = 0.9997 for Folin-Ciocalteu, and *y* = 0.0099*x* + 0.0547, *R* ^2^ = 0.9916 for Fast Blue BB.

#### 2.4.2. HPLC Apparatus and Chemicals

The analysis was performed by a PerkinElmer Series 200 high-performance liquid chromatography coupled to diode array detector (HPLC-DAD) consisting of a quaternary pump, online degasser, and autosampler. Data were processed using the TotalChrom Workstation software. The injection volume was 10 *μ*L. A 5 *μ*m PerkinElmer Brownlee Choice C-18, 4.6 × 150 mm analytical column was used. The column was maintained at 24 ± 2°C (room temperature).

#### 2.4.3. Procedure

The analysis was conducted according to Costa et al. (2011) [11]. A MEVM sample (0.01 mg/10 mL) was filtered with 0.45 *μ*m polyester filters and the solvent system consisted of (A) acetonitrile and (B) 1% acetic acid, adjusted to pH 3.0. Solvents were provided at a flow rate of 1.0 mL min^−1^ using the following gradient: 0–30 min, linear change from A-B (5 : 95 v/v) to A-B (20 : 80 v/v); 30–40 min, isocratic A-B (20 : 80 v/v). Detection was monitored at 340 nm and samples were performed in duplicate.

### 2.5. Pharmacological Procedures

#### 2.5.1. Animals

Male C57BL/6LDLr-null mice were used in the experiments. Animals were provided by the* Centro Multidisciplinar para Investigação Biológica na Área da Ciência de Animais de Laboratório* (CEMIB, UNICAMP, Campinas/SP, Brazil) and were kept at temperature- and light-controlled room (22 ± 2°C; 12 h light/dark cycle) with free access to water and food. All experimental procedures adopted in this study were previously approved by the Institutional Ethics Committee of* Universidade Paranaense* (UNIPAR, Brazil; authorization number 24374/2013).

#### 2.5.2. Experimental Groups and Hyperlipidemia and Atherogenesis Induction

Dyslipidemia and atherogenesis were induced by administration of commercial high fat diet (HFD) for 6 weeks (RHOSTER Ind. Com. LTDA, São Paulo/SP, Brazil). The basic formulation contained a mixture of cornstarch, DL-methionine, lard, casein, calcium carbonate, cellulose, cholesterol, soybean oil, AIN-76 mineral premix, AIN-93 vitamin premix, sucrose, and tert-butylhydroquinone. HFD was offered* ad libitum* to groups of mice from 3 months of age. The animals were used in the study after confirmation of hypercholesterolemia.

Two weeks after starting the diet, the animals were randomly assigned to one of six experimental groups (*n* = 7) as follows:

1. negative control: C57BL/6LDLr-null mice receiving standard commercial diet and treated for three weeks with vehicle;
2. positive control: C57BL/6LDLr-null mice receiving HFD and treated for three weeks with vehicle;
3. MEVM30: C57BL/6LDLr-null mice receiving HFD and treated for three weeks with* Vitex megapotamica* extract at dose of 30 mg/kg;
4. MEVM100: C57BL/6LDLr-null mice receiving HFD and treated for three weeks with* Vitex megapotamica* extract at dose of 100 mg/kg;
5. MEVM300: C57BL/6LDLr-null mice receiving HFD and treated for three weeks with* Vitex megapotamica* extract at dose of 300 mg/kg;
6. SIMV 40: C57BL/6LDLr-null mice receiving HFD and treated for three weeks with simvastatin at dose of 40 mg/kg.

*Vitex megapotamica* extract was orally administered at the doses above, once a day, for three weeks, starting from the 2nd week of treatment with HFD. All parameters described below were measured during or after the treatment period.

#### 2.5.3. Systolic Blood Pressure (SBP)

To measure blood pressure, conscious mice were comfortably adapted in media for rodents. After 103 min of warm-up period, systolic blood pressure was obtained via tail-cuff by using computerized system from AD Instruments (Castle Hill, Australia). Ten preliminary cycles were performed to allow mice to adapt to the inflating cuff. Subsequently, a minimum of five cycles of systolic blood pressure measurements were obtained for each animal. The mean systolic blood pressure of each animal was then calculated. Systolic blood pressure measurements were carried out on a weekly basis from the beginning of experiments for 5 weeks.

#### 2.5.4. Diuretic Activity and Renal Function Assessment

The diuretic activity was determined on a weekly basis from the beginning of experiments for 5 weeks in all experimental groups according to method previously described [12]. Animals were fasted overnight with free access to water. Each animal was placed in an individual metabolic cage (Type 304 Stainless steel, Tecniplast, Italy) for a period of 24 h for environmental adaptation. Before experiment, all animals received physiological saline (0.9% NaCl) in an oral dose of 0.5 mL/10 g to provide uniform water and salt load. Urine was collected in a graduated cylinder and the volume was recorded at 2 h intervals for 8 h. Cumulative urine excretion was calculated in relation to body weight and expressed as mL/10 g. Electrolyte concentrations (Na^+^, K^+^, Cl^−^, and HCO_3_^−^), pH, density, and conductivity were estimated using urine samples from each mouse collected at the end of the experiment (8 h).

*(1) Analytical Procedures*. For serum analysis, blood samples were collected in conical tubes after decapitation at the end of the experimental period. Serum was obtained by centrifugation (2000 rpm, 10 minutes, 4°C) and stored at −20°C until analysis. Plasmatic and urinary Na^+^ and K^+^ levels were quantified by flame photometry (Quimis model Q398112). Cl^−^ and HCO_3_^−^ concentrations were quantified by titration. pH and conductivity were directly determined on fresh urine samples using Q400MT pHmeter and Q795M2 conductivity meter (Quimis Instruments, Brazil), respectively. Density was estimated by weighing with a Mettler AE163 (±0.1 mg) analytical scale on urine volume measured with Nichiryo micropipette. Plasma urea, creatinine, and total protein levels were determined by enzymatic method using BM/Hitachi 912 automated analyzer (Cobas Mira, Roche, Indianapolis, USA).

#### 2.5.5. Serum Lipid Profile and Atherogenic Index (AIS)

Triglyceride (TG), total cholesterol (TC), and high-density lipoprotein cholesterol (HDL-C) levels were measured using biochemical test kits with semiautoanalyzer by colorimetric method at the end of treatments. The AIS, which is the measure of the extent of atherosclerotic lesions based on serum lipids, was determined in all groups. The atherogenic index is calculated using the following formula: AIS = TC/HDL [13].

#### 2.5.6. Determination of Serum Nitrate/Nitrite (NO_*x*_)

The plasma nitrite concentration was determined by enzymatically reducing nitrate using nitrate reductase enzyme at the end of the experimental period [14]. Plasma samples were collected from animals, deproteinized with zinc sulfate (30 mmol), and diluted 1 : 1 with Milli-Q water. For conversion of nitrate into nitrite, samples were incubated at 37°C for 2 hours in the presence of nitrate reductase expressed in* E. coli*. After the incubation period, samples were centrifuged (800 g, 10 minutes) to remove bacteria. Then, 100 *μ*L of supernatant was mixed with equal volume of Griess reagent (1% sulfanilamide in 10% phosphoric acid/0.1% alpha-naphthyl-ethylenediamine in Milli-Q water) in a 96-well plate and read at 540 nm in a plate reader. Standard nitrite and nitrate curves (0–150 mM) were performed simultaneously.

#### 2.5.7. Evaluation of Lipid Peroxidation

The thiobarbituric acid (TBARS) levels were measured using commercial assay kits (Nanjing Jiancheng Institute, Nanjing, China) on a spectrophotometer (DU7400, Beckman Co., Fullerton, CA, USA), according to manufacturer's instruction. This method was used to obtain a spectrophotometric measurement of the color produced during the reaction of thiobarbituric acid and malondialdehyde (an indicator of peroxidation of polyunsaturated fatty acids in cell membranes subsequent to reactions with reactive oxygen species) at 535 nm. The TBARS level was expressed as mmol/L.

### 2.6. Statistical Analyses

Results are expressed as mean ± standard error of the mean of seven animals in each group. Statistical evaluation was carried out using analysis of variance (ANOVA) followed by Bonferroni's test. A *P* value less than 0.05 was considered statistically significant. Graphs were drawn and statistical analysis was carried out using GraphPad Prism version 5.0 for Mac OS X (GraphPad Software, San Diego, CA, USA).

## 3. Results

### 3.1. Phytochemical Analysis

**Figure 1:**
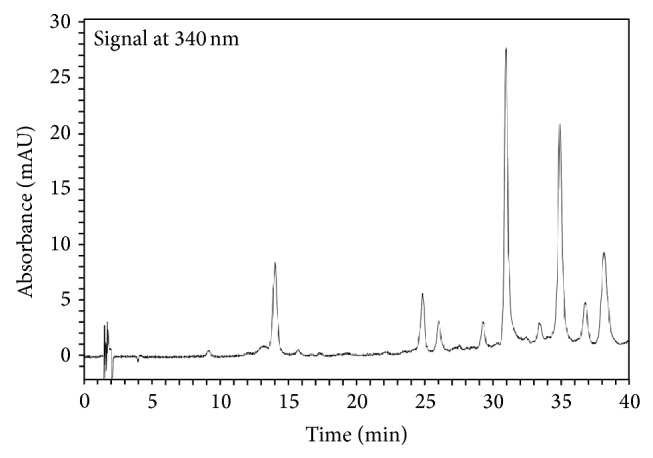
Representative HPLC-DAD chromatogram of* Vitex megapotamica *methanolic extract (MEVM). Chromatographic conditions are specified in Section 2.

The methanolic extract showed strongly positive reaction with flavonoids and saponins and low-intensity reaction with tannins and catechins. MEVM analysis (100 *μ*g/mL) resulted in mean GAE values of 40 and 64 *μ*g using Folin-Ciocalteu and Fast Blue BB methods. Furthermore, the fingerprint obtained from MECS showed five peaks with absorbance at 340 nm (Figure 1) compatible with polar flavonoid glycosides.

### 3.2. MEVM Prevents Progressive Increase in SBP in LDLr-Null Mice

**Figure 2:**
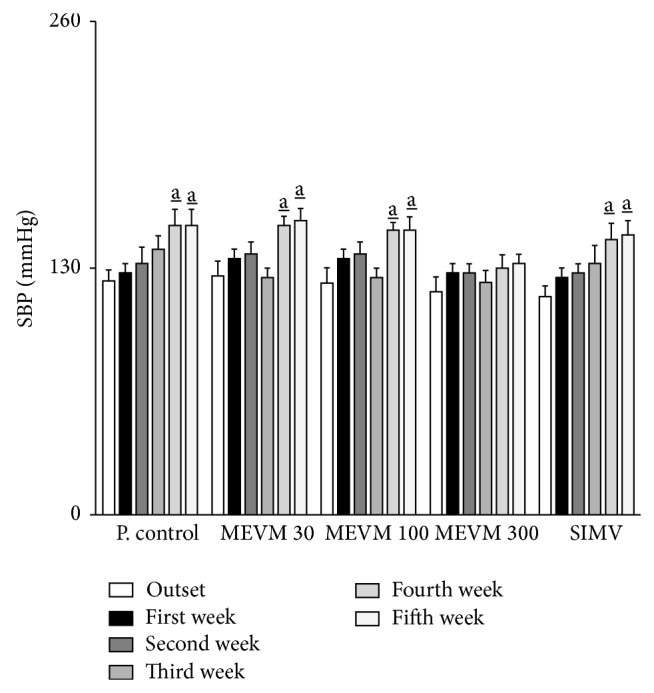
Effects of the treatment with methanolic extract of* Vitex megapotamica* (MEVM) on systolic blood pressure of C57BL6 LDLr-null mice. Groups of mice (*n* = 7) were treated for 5 weeks with commercial atherogenic diet. At the end of the second week the animals received varying doses of MEVM (30–300 mg/kg) or simvastatin (40 mg/kg) for three more weeks. Systolic blood pressure measurements were carried out on a weekly basis from the beginning of experiments for 5 weeks. Each bar represents the mean and the vertical lines show the SEM. $\underline{\text{a}}$ denotes significance levels compared to the beginning of the experiments (outset). Two-way ANOVA followed by Bonferroni test (Pa_<0.05).

In C57BL/6 LDLr-null mice, the administration of atherogenic diet alone was able to increase SBP by approximately 20% after 4 weeks of treatment. On the other hand, in animals receiving MEVM (300 mg/kg) but not simvastatin, SBP was not significantly altered when compared to the beginning of treatments (Figure 2).

### 3.3. MEVM Prevents Progressive Renal Impairment and Increases Urinary Sodium Excretion in LDLr-Null Mice

**Figure 3:**
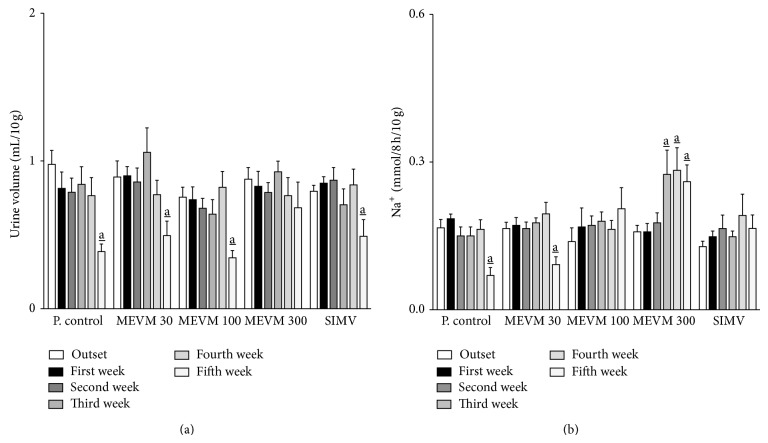
Treatment with methanolic extract of* Vitex megapotamica *(MEVM) preserves urine volume (a) and increases renal sodium excretion (b) in C57BL6 LDLr-null mice. Groups of mice (*n* = 7) were treated for 5 weeks with commercial atherogenic diet. At the end of the second week the animals received varying doses of MEVM (30–300 mg/kg) or simvastatin (40 mg/kg) for three more weeks. Renal function was determined out on a weekly basis from the beginning of experiments for 5 weeks. Each bar represents the mean and the vertical lines show the SEM. $\underline{\text{a}}$ denotes significance levels compared to the outset of experiments. Two-way ANOVA followed by Bonferroni test (Pa_<0.05).

**Figure 4:**
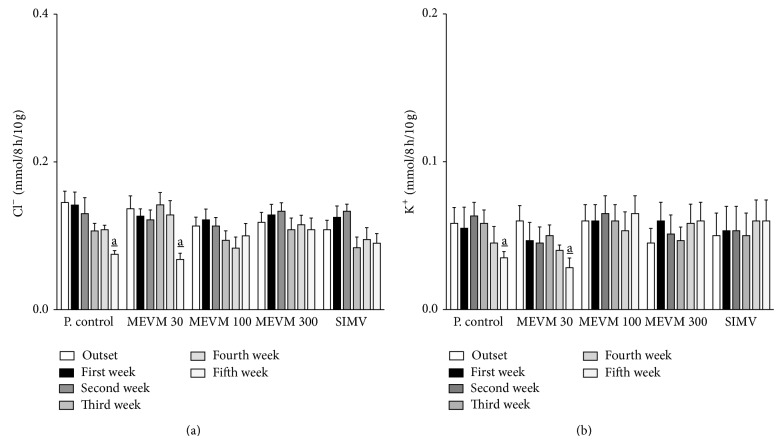
HEVM-treatment prevents the reduction of renal excretion of chloride (a) and potassium (b) in C57BL6 LDLr-null mice. Groups of mice (*n* = 7) were treated for 5 weeks with commercial atherogenic diet. At the end of the second week the animals received varying doses of MEVM (30–300 mg/kg) or simvastatin (40 mg/kg) for three more weeks. Electrolytes excretion was determined out on a weekly basis from the beginning of experiments for 5 weeks. Each bar represents the mean and the vertical lines show the SEM. $\underline{\text{a}}$ denotes significance levels compared to the outset of experiments. Two-way ANOVA followed by Bonferroni test (Pa_<0.05).

Urinary volume, density, conductivity, sodium, chloride, and potassium excretion values before treatment (outset) were, respectively, 0.97 ± 0.09 mL/10 g, 1005 ± 0.6 g/L, 15.75 ± 0.11 mS/cm, 0.16 ± 0.02, 0.14 ± 0.01, and 0.06 ± 0.01 mmol/8 h/10 g. The administration of atherogenic diet in LDL-null mice was able to induce an expressive reduction in urinary volume (0.38 ± 0.05 mL/10 g; *P* < 0.05), urinary conductivity (15.75 ± 0.11 mS/cm; *P* < 0.05), and sodium (0.06 ± 0.01 mmol/8 h/10 g; *P* < 0.05), chloride (0.07 ± 0.01 mmol/8 h/10 g; *P* < 0.05), and potassium (0.03 ± 0.01 mmol/8 h/10 g; *P* < 0.05) excretion values after 5 weeks of treatment (Table 1; Figures 3 and 4). Furthermore, urinary density, serum creatinine, and urea values were significantly increased, while plasmatic total protein shows an expressive reduction (~50%) at the end of the experimental period (Table 1; Figures 6(a)–6(c)). On the other hand, treatment with MEVM (300 mg/kg) was able to maintain urinary and plasmatic parameters with similar values in negative control groups (Tables 1 and 2; Figures 3, 4, and 6(a)–6(c)). Moreover, MEVM (300 mg/kg) increased the urinary sodium excretion (~65%) after acute treatment, while maintaining the natriuretic effect throughout the experimental period (Figure 3(b)). Animals treated with simvastatin did not show sustained diuretic or natriuretic effect; however, they presented a renoprotective effect in the other renal function parameters. The pH values were not affected by any of treatments.

### 3.4. MEVM Induces Lipid-Lowering and Atheroprotective Effects in Swiss and LDLr-Null Mice

**Figure 5:**
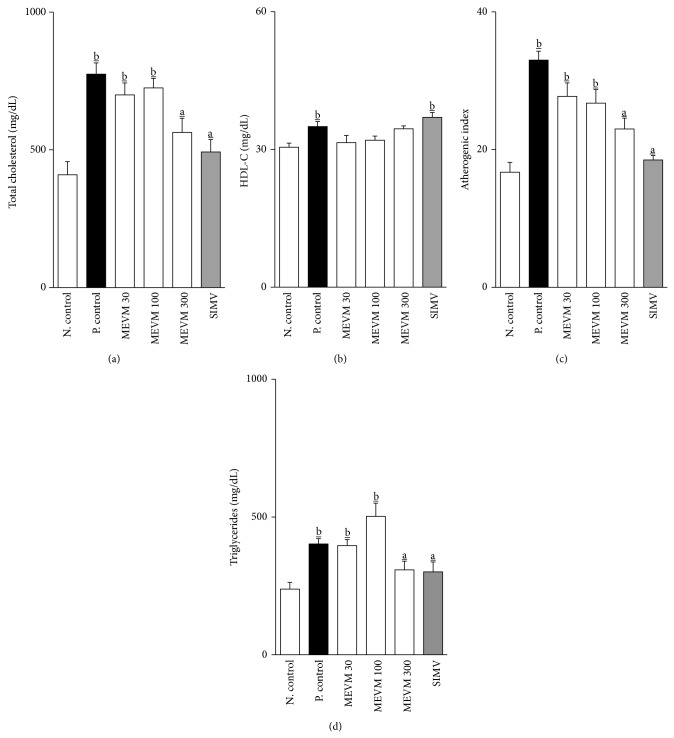
Prolonged MEVM administration induces lipid-lowering and atheroprotective effects ((a)–(d)) in C57BL6 LDLr-null mice. Groups of mice (*n* = 7) were treated for 5 weeks with commercial atherogenic diet. At the end of the second week the animals received varying doses of MEVM (30–300 mg/kg) or simvastatin (40 mg/kg) for three more weeks. All analyses were performed at the end of the experimental period (5 weeks). Values are expressed as mean ± SEM (*n* = 7 in each group) in comparison to the positive control (a; *P* < 0.05) or negative control (b; *P* < 0.05) using one-way ANOVA followed by Bonferroni test.

Serum total cholesterol (TC), high-density lipoprotein cholesterol (HDL-C), and triglycerides (TG) values are presented in Figure 5. Serum TC in LDLr-null mice (baseline 409 ± 47 mg/dL) increased to 775 ± 40 mg/dL in positive controls after 7 weeks of HFD. At the same time, values shown by SIMV treated mice were significantly lowered to 492 ± 44 (*P* < 0.05). Similarly, a significant reduction in TC levels was also evident in HFD mice treated with* Vitex megapotamica* extracts. In animals that received MEVM (300 mg/kg), TC reduced to levels of 563 ± 51 mg/dL (*P* < 0.05). In addition, HDL-C levels were not significantly changed after treatment with all MEVM doses. Additionally, triglyceride levels were significantly reduced when compared to positive control group (positive control: 402 ± 19; MEVM 300: 308 ± 26; *P* < 0.05) after prolonged MEVM administration. Triglycerides levels measured in MEVM-treated groups were close to those found in SIMV group.

The AIS was significantly higher in HFD positive controls after 5 weeks of experiment. Treatment with HEMV (300 mg/kg) provided a significant reduction in AIS when compared to HFD positive control groups (HEVM: 22.0 ± 1.48; positive control: 31.0 ± 1.12; *P* < 0.05). The values found at the dose of 300 mg/kg were close to those found in SIMV or negative control groups (Figure 5(c)).

### 3.5. Antioxidant Activity of MEVM in High Fat Mice

**Figure 6:**
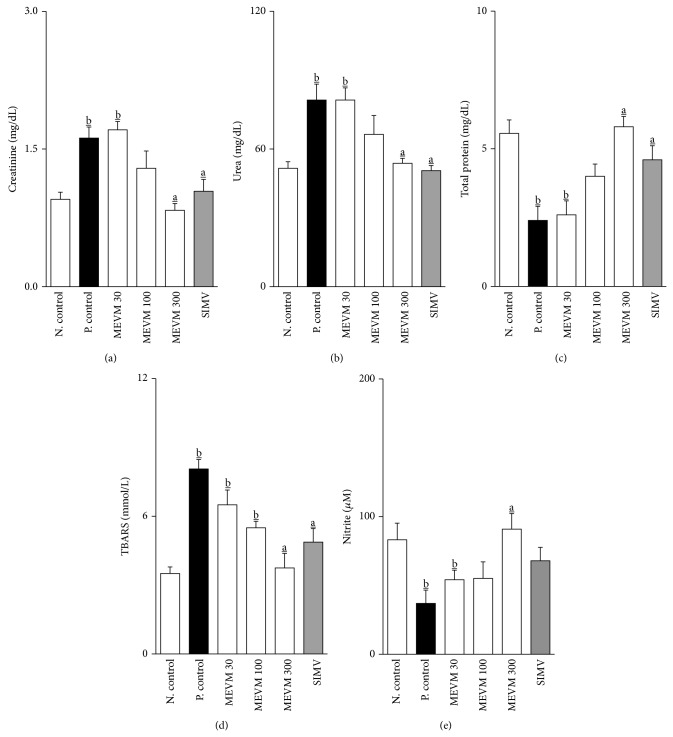
MEVM administration prevents alterations in biomarkers of renal function ((a)–(c)) and shows significant antioxidant activity ((d)-(e)) in C57BL6 LDLr-null mice. Groups of mice (*n* = 7) were treated for 5 weeks with commercial atherogenic diet. At the end of the second week the animals received varying doses of MEVM (30–300 mg/kg) or simvastatin (40 mg/kg) for three more weeks. All analyses were performed at the end of the experimental period (5 weeks). Values are expressed as mean ± SEM (*n* = 7 in each group) in comparison to the positive control (a; *P* < 0.05) or negative control (b; *P* < 0.05) using one-way ANOVA followed by Bonferroni test.

The antioxidant effect of MEVM was estimated by TBARS and nitrite measurement (marker of nitric oxide bioavailability). In LDL-null mice, MEVM was able to reduce the TBARS levels by approximately 50%. Additionally, the nitrite levels increased by approximately 85% when compared to positive control groups (Figures 6(d)-6(e)).

## 4. Discussion

In recent years, atherosclerosis has received notable attention for triggering significant changes in renal function because glomerular cells mimic some of the characteristics of cells in the vessel wall; atherosclerosis and glomerulosclerosis are postulated as comparable processes. Furthermore, it is well established that renal injury is aggravated by HFD and atherosclerosis. On the other hand, progressive deterioration of the renal function may lead to dyslipidemia, which can induce production of reactive oxygen species (ROS) and activate proinflammatory and fibrogenic factors, leading to vascular endothelial cell dysfunction, favoring the development of atherosclerosis. Therefore, kidney can be a villain or a victim during atherogenesis [15, 16].

Atherosclerotic changes in the renal artery are evident in 50% of patients with previous atherosclerotic disease and in 6.8% of adults aged 65 years or older; it can induce significant reduction in renal function [17]. Evidence indicates that increased oxidative stress and inflammation may mediate a large part of the effects of risk factors on the kidney. In the renal arteries, as well as in other arteriolar beds, traditional cardiovascular risk factors such as HFD may promote endothelial damage and induce oxidative stress and inflammation that might result in reduction of nitric oxide (NO) production and endothelial dysfunction. Among other effects, these changes may lead to impaired endothelium-mediated relaxation in arterial segments, thereby increasing the risk of high blood pressure, smooth muscle cell proliferation, atherosclerosis progression, and renal injury [18].

When considering an ideal model to study atherosclerosis-induced renal failure, several difficulties are found. One of them is the fact that rodents, in general, are very resistant to HFD-induced renal injury. In this sense, a mice strain highly sensitive to atherosclerotic lesions was used in this study (LDLr-null). This mice strain mimics atherosclerotic lesions and other resulting changes in a similar way to what occurs to humans. Thus, after 5 weeks of HFD, all mice in our study showed dyslipidemia and significant increase in lipid peroxidation, accompanied by a reduction of nitric oxide bioavailability with consequent elevation of SBP. As hypertension, dyslipidemia and oxidative stress can directly damage the kidney and by promoting intrarenal atherogenesis even in the absence of obstructive lesions in the renal artery, important decrease in the renal function was also observed, with significant reduction in urine volume and renal electrolyte excretion, accompanied by significant changes in serum creatinine, urea, and total protein levels.

Although renal changes are evident, treatment for 3 weeks with HEMV (300 mg/kg) was able to prevent reductions in renal function observed in all untreated animals. In this case, urine elimination, density, conductivity, and renal excretion of potassium, chloride, and bicarbonate remained with values close to those obtained for control animals. In addition, treatment with HEMV was able to normalize serum creatinine, urea, and total protein, important biochemical markers of renal function. Regarding the renoprotective effect observed, what most attracted attention was the fact that HEMV causes an expressive urinary sodium excretion, significantly higher than in all experimental groups. Possibly, this natriuretic effect, along with the other activities, can be corroborated for the reduction of cardiovascular changes, especially in reducing blood pressure levels observed in the last two weeks of study.

In addition to activities directly renoprotective, MEVM treatment was able to reduce the TC levels in LDLr-null mice without affecting the elevation of HDL levels. Furthermore, a significant reduction in serum triglyceride was also observed, as previously reported [7]. Treatment with the highest dose of MECS showed significant reduction in the atherosclerotic index serum (AIS), with potential cardiovascular and renoprotective effect of this species. Together, serum lipids and AIS may indicate the deposition of foam cells or fatty infiltration in heart, coronaries, and kidney, which means a greater likelihood of oxidative damage in these target organs [13]. In addition, the hypolipidemic activity of MEVM was favorably compared to the standard drug (simvastatin) especially at the highest dose (300 mg/kg).

Another very significant finding in this study was the reduction in the oxidative stress, which plays a critical role in the endothelium dysfunction and in the LDL oxidation, characterizing the initial step of changes that lead to atherosclerosis and renal injury. Oxidation biomarkers such as TBARS are markedly increased during HFD consumption and may represent independent indicators of risk for patients with atherosclerosis. The fact that MECS increased NO levels (measured as nitrite), it may be able to reduce oxidative insults and lipid peroxidation caused by HFD. On the other hand, the antioxidant properties of the secondary metabolites of* Vitex megapotamica* extract (mainly polyphenolic compounds) may also have contributed to elevate NO levels and prevent SBP elevation.

A frequent question in our study was about which HEMV components could be involved in the activities observed. Phytochemical analysis of HEMV revealed the predominant presence of flavonoid glycosides and other polyphenols. Currently, it is well established that flavonoids and other polyphenols may have antioxidant, diuretic, natriuretic, and antihypertensive activity, suggesting several cardioprotective effects, including decreased oxidation of LDL cholesterol and endothelial damage [9, 19, 20]. Although these compounds proactively collaborate to prevent the onset of atherosclerosis, the fact that the pharmacological effects of HEVM may be due to several other secondary metabolites acting in a coordinated manner, but in independent molecular targets cannot be ruled out. In fact, the idea of a phytocomplex with pleiotropic effects is now widely accepted in communities around the world, especially in Brazil, due to the possibility of using herbal drugs as adjuvant in different renal and cardiovascular diseases.

## 5. Conclusion

In short, we showed that MEVM might present compounds responsible for reducing serum lipids and oxidative stress when orally administered. In addition, it was able to prevent renal injury induced by HFD in LDLr-null mice. Despite all the evidence that phenolic compounds can contribute to these effects, other metabolites that may be operating in an integrated manner in the cardioprotective effects of this specie should not be excluded. Moreover, the data reported here collaborate with preclinical investigations of this species and support the potential of MEVM from* Vitex megapotamica* as candidate for phytomedicine to be used in cardiovascular or renal diseases where antioxidant, natriuretic, and hypolipidemic effects are desired.

## Figures and Tables

**Table 1:** Effects of the administration of the methanolic extract of *Vitex megapotamica* (MEVM) and simvastatin (SIMV) on urinary density from LDLr-null mice.

Group	Density (g/L)					
	Outset	Week 1	Week 2	Week 3	Week 4	Week 5
N. control	1004 ± 0.3	1005 ± 0.9	1004 ± 0.7	1006 ± 0.7	1004 ± 0.9	1005 ± 0.6
P. control	1005 ± 0.6	1006 ± 1.0	1007 ± 0.9	1007 ± 0.8	1008 ± 1.1	1011 ± 1.0^b^
MEVM (30)	1006 ± 0.8	1007 ± 0.9	1005 ± 0.6	1006 ± 0.9	1007 ± 0.8	1007 ± 0.9
MEVM (100)	1005 ± 0.5	1004 ± 0.6	1005 ± 0.4	1006 ± 0.7	1005 ± 0.6	1006 ± 0.5^a^
MEVM (300)	1006 ± 0.8	1006 ± 0.7	1004 ± 0.7	1005 ± 0.9	1006 ± 0.8	1006 ± 0.7^a^
SIMV (40)	1005 ± 0.5	1004 ± 0.6	1005 ± 0.8	1005 ± 0.6	1006 ± 0.6	1005 ± 1.3^a^

Values are expressed as mean ± SEM (*n* = 7 in each group) in comparison to the positive control (^a^ *P* < 0.05) or negative control (^b^ *P* < 0.05) using one-way ANOVA followed by Bonferroni test.

**Table 2:** Effects of the administration of the methanolic extract of *Vitex megapotamica* (MEVM) and simvastatin (SIMV) on urinary conductivity from LDLr-null mice.

Group	Conductivity (mS/cm)					
	Outset	Week 1	Week 2	Week 3	Week 4	Week 5
N. control	15.56 ± 0.11	15.36 ± 0.12	15.16 ± 0.22	14.26 ± 0.19	14.99 ± 0.21	15.01 ± 0.22
P. control	15.75 ± 0.11	14.99 ± 0.20	14.88 ± 0.18	15.01 ± 0.15	15.17 ± 0.19	12.29 ± 0.18^b^
MEVM (30)	15.46 ± 0.28	15.36 ± 0.19	15.94 ± 0.20	16.14 ± 0.21	15.21 ± 0.25	13.33± 0.19^b^
MEVM (100)	16.06 ± 0.23	16.10 ± 0.13	15.57 ± 0.18	15.27 ± 0.15	15.27 ± 0.15	15.57 ± 0.17
MEVM (300)	15.30 ± 0.22	15.20 ± 0.20	15.70 ± 0.19	17.96 ± 0.15^a,b^	17.16 ± 0.17^a,b^	17.99 ± 0.18^a,b^
SIMV (40)	16.29 ± 0.64	15.96 ± 0.20	14.99 ± 0.18	14.99 ± 0.18	15.01 ± 0.19	15.81 ± 0.19

Values are expressed as mean ± SEM (*n* = 7 in each group) in comparison to the positive control (^a^ *P* < 0.05) or negative control (^b^ *P* < 0.05) using one-way ANOVA followed by Bonferroni test.
